# Intestinal Damage in COVID-19: SARS-CoV-2 Infection and Intestinal Thrombosis

**DOI:** 10.3389/fmicb.2022.860931

**Published:** 2022-03-22

**Authors:** Xiaoming Wu, Haijiao Jing, Chengyue Wang, Yufeng Wang, Nan Zuo, Tao Jiang, Valerie A. Novakovic, Jialan Shi

**Affiliations:** ^1^Department of Hematology, The First Hospital, Harbin Medical University, Harbin, China; ^2^Department of General Surgery, The First Hospital, Harbin Medical University, Harbin, China; ^3^Department of Research, VA Boston Healthcare System, Harvard Medical School, Boston, MA, United States; ^4^Department of Medical Oncology, Dana-Farber Cancer Institute, Harvard Medical School, Boston, MA, United States

**Keywords:** COVID-19, blood transmission, intestinal infection, intestinal thrombosis, antithrombotic therapy

## Abstract

The intestinal tract, with high expression of angiotensin-converting enzyme 2 (ACE2), is a major site of extrapulmonary infection in COVID-19. During pulmonary infection, the virus enters the bloodstream forming viremia, which infects and damages extrapulmonary organs. Uncontrolled viral infection induces cytokine storm and promotes a hypercoagulable state, leading to systemic microthrombi. Both viral infection and microthrombi can damage the gut–blood barrier, resulting in malabsorption, malnutrition, and intestinal flora entering the blood, ultimately increasing disease severity and mortality. Early prophylactic antithrombotic therapy can prevent these damages, thereby reducing mortality. In this review, we discuss the effects of SARS-CoV-2 infection and intestinal thrombosis on intestinal injury and disease severity, as well as corresponding treatment strategies.

## Introduction

COVID-19 has become a worldwide pandemic causing widespread illness and mortality. SARS-CoV-2 mainly infects the respiratory tract through attachment to angiotensin-converting enzyme 2 (ACE2) receptors (Lan et al., 2020). ACE2 is also highly expressed on intestinal epithelial cells, allowing SARS-CoV-2 to infect the intestinal tract (Xiao et al., 2020a). Recent meta-analyses show that 48%–54% of fecal samples from COVID-19 patients have tested positive for viral RNA, and 15%–17% of patients have gastrointestinal (GI) symptoms (Cheung et al., 2020; Mao et al., 2020; Sultan et al., 2020). Additionally, live virus can be isolated from fecal samples of COVID-19 patients (Wang et al., 2020). Some studies have proposed fecal–oral transmission as the cause of intestinal infection (Guo et al., 2021). However, direct evidence for fecal–oral transmission is still lacking. Meanwhile, the virus has been detected in the blood of both symptomatic and asymptomatic patients (Chang et al., 2020), and disseminated virus could infect extrapulmonary organs (Jacobs and Mellors, 2020). Thus, the potential that intestinal infection occurs *via* blood transmission should be carefully considered.

Pulmonary infection triggers cytokine storm and induces a prothrombotic state (McFadyen et al., 2020; Moore and June, 2020). Venous and arterial thrombosis are common in COVID-19 (Moore and June, 2020). Systematic reviews estimate that 14%–31% of in-hospital patients develop a clinically apparent thrombotic event (Suh et al., 2021; Tan et al., 2021), while autopsy reports show a high prevalence of microthrombi in multiple organs, including lung, heart, liver, kidney, and gastrointestinal tract (Bradley et al., 2020; Polak et al., 2020). A cohort study showed that COVID-19 patients with intestinal ischemia had markedly elevated D-dimer levels and poor outcomes (Norsa et al., 2020). Additionally, recent studies have shown that mesenteric thrombosis often results in intestinal resection and significantly increases mortality (Bhayana et al., 2020; El Moheb et al., 2020). Therefore, it is essential to outline the mechanisms of intestinal thrombosis and its contribution to intestinal damage and disease progression.

In this review, we discuss blood transmission as a potential route for intestinal infection. We then summarize the characteristics and mechanism of intestinal thrombosis formation in COVID-19. Next, we focus on the effects of intestinal infection and thrombosis on intestinal damage and disease severity. Finally, we discuss therapeutic strategies to prevent intestinal damage.

## Gastrointestinal Symptoms and SARS-CoV-2 Infection

Multiple studies have reported GI symptoms in COVID-19 patients, including diarrhea, nausea, vomiting, anorexia, and abdominal pain (Cheung et al., 2020; Mao et al., 2020; Sultan et al., 2020). According to a meta-analysis comprising 10,890 COVID-19 patients, the pooled prevalence estimates of GI symptoms were: diarrhea (7.7%), nausea or vomiting (7.8%), and abdominal pain (2.7%; Sultan et al., 2020) with 10% of these patients reporting GI symptoms as being their initial symptoms (Cheung et al., 2020). These data indicate potential gastrointestinal infection by SARS-CoV-2, which is reported to infect and replicate in epithelial cells of human small intestinal organoids (Zang et al., 2020). Both viral nucleocapsid proteins and viral particles have been detected in infected patient intestinal biopsies (Livanos et al., 2021). Additionally, SARS-CoV-2 RNA and live virus can be found in the stool of patients (Wang et al., 2020). More importantly, SARS-CoV-2 subgenomic mRNA is transcribed in actively replicating cells and has been detected in fecal samples (Wölfel et al., 2020). Further, rectal viral shedding persists for longer than that of the respiratory system (Zhao et al., 2020). All these data demonstrate that SARS-CoV-2 directly infects and replicates in intestinal epithelial cells of patients.

## Intestinal Infection and Transmission Routes

**Figure 1:**
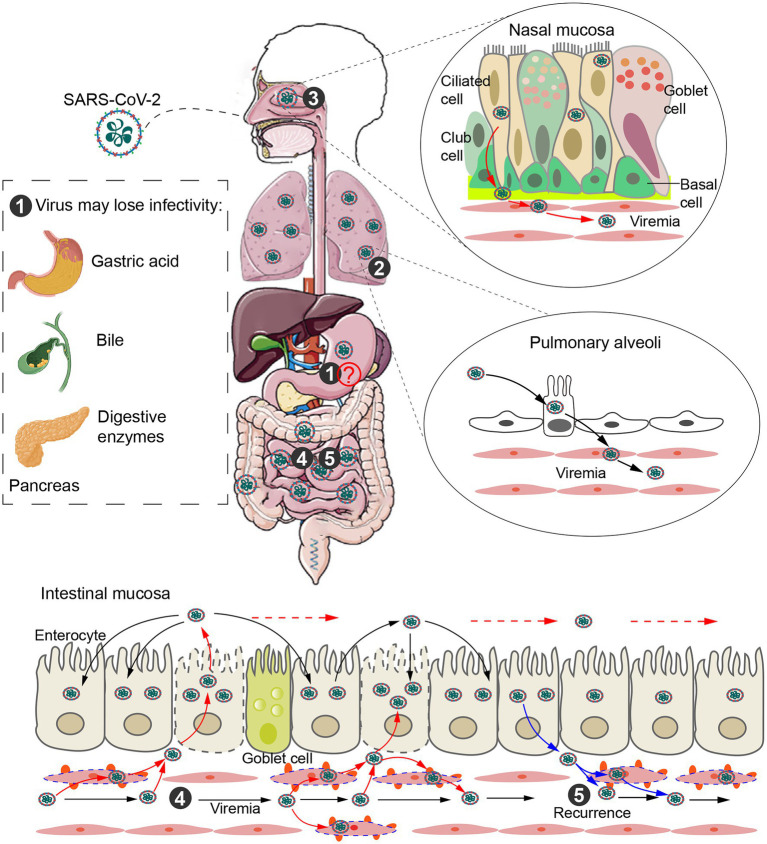
Intestinal infection and transmission routes. ① Direct evidence for fecal–oral transmission is still lacking. SARS-CoV-2 may be unable to enter the small intestine from the stomach due to gastric acid, bile and digestive enzymes. ② SARS-CoV-2 released from type II alveolar cells infects alveolar capillary endothelial cells (ECs). The virus replicates in ECs and is released into the blood to form viremia. ③ SARS-CoV-2 is released from infected ciliary cells of the nasal cavity and breaks through the basement membrane, infecting the vascular ECs and eventually entering circulation. ④ Blood transmission after alveolar or nasal infection is a potential route of intestinal infection. Eventually, SARS-CoV-2 is released into the gut and infects surrounding intestinal epithelial cells along the intestinal tract. ⑤ SARS-CoV-2 in the gut can also enter the capillaries and cause viremia, leading to recurrence of disease.

With the deepening understanding of COVID-19, GI symptoms have been recognized as early signs of the disease. The high expression of ACE2 in the GI tract, isolation of live virus from fecal samples, and a subset of patients presenting with only GI symptoms seem to suggest fecal–oral transmission. However, problems with the feasibility of this mode of transmission remain. First, studies have shown that SARS-CoV-2 loses infectivity in simulated gastric acid within 10 min (Chan et al., 2020; Zang et al., 2020; Zhong et al., 2020). Secondly, SARS-CoV-2, as an enveloped virus, is largely unable to withstand the detergent effect of bile salts and the activity of digestive enzymes in the duodenum (Figure 1). Although some studies have suggested that highly viscous mucus in the gastrointestinal tract protects SARS-CoV-2, allowing the virus to retain its infectivity (Guo et al., 2021; Zhang H. et al., 2021), there is still a lack of direct evidence. Bushman et al. (2019) had previously investigated the links between the structures of viruses and routes of transmission and found a strong association between fecal–oral transmission and the absence of a lipid envelope. Lastly, although some studies have isolated intact viruses from feces (Wang et al., 2020; Zhang Y. et al., 2020; Zhou et al., 2020; Xiao et al., 2020b), most of them have not further confirmed the infectivity of these viruses (Wang et al., 2020; Zhang Y. et al., 2020; Xiao et al., 2020b). Zhou et al. (2020) confirmed viral propagation by RT-PCR, but only in a single fecal sample. Previous research has shown that SARS-CoV-2 is completely inactivated in simulated human colonic fluid over the course of 24 h, which may explain the sporadic detection of infection-active SARS-CoV-2 from feces samples.

Several lines of evidence suggest that SARS-CoV-2 may infect the intestinal tract *via* the bloodstream. Deng et al. (2020) detected SARS-CoV-2 RNA in anal swabs from intratracheally but not intragastrically infected rhesus macaques, suggesting blood transmission. Indeed, SARS-CoV-2 RNA has been detected in blood and urine samples of patients (Wang et al., 2020). The virus can also be detected in multiple organs (including heart, brain, and kidney) and is associated with organ injury, indicating that the virus can reach and infect extrapulmonary organs (Puelles et al., 2020). Another study showed that SARS-CoV-2 viremia was associated with intestinal damage, independent of disease severity (Li Y. et al., 2021). Thus, blood transmission could be the cause of intestinal infection. Specifically, SARS-CoV-2 replicating in alveolar epithelial cells and capillary ECs is released into the bloodstream and infects new vascular ECs. The capillary network is then the main route by which the virus enters and infects extrapulmonary organs. The extensive surface area of intestinal capillaries makes intestinal epithelial cells more susceptible to infection than other extrapulmonary organs. Following infection of intestinal capillaries, SARS-CoV-2 is released into the gut and infects surrounding intestinal epithelial cells along the intestinal tract (Figure 1). Once established in the gut, SARS-CoV-2 can also reenter the capillaries, potentially leading to recurrence of disease. Consistent with this, in patients who experienced recurrence, the phylogenetic analysis of infection samples has shown that recurrent virus evolves from the original parent virus (Hu et al., 2020).

Additionally, SARS-CoV-2 RNA can also be detected in the blood and urine of asymptomatic patients, suggesting a second pathway to viremia through the nasal cavity (Chang et al., 2020; Hasanoglu et al., 2021). The abundant blood vessels, thin mucous membrane, and higher levels of ACE2 (Huang et al., 2021) make it possible for the virus to initiate viremia from the nasal cavity. Specifically, SARS-CoV-2 is released from infected ciliary cells of the nasal cavity and breaks through the basement membrane, infecting the vascular ECs and eventually entering circulation (Figure 1). Blood transmission after nasal infection is therefore another potential route of intestinal infection.

## Intestinal Damage, Malnutrition, and Poor Outcomes

A recent study has shown that a fecal sample positive for SARS-CoV-2 RNA at any time during hospitalization was associated with higher mortality [HR: 3.4 (1.2–9.9); Das Adhikari et al., 2021]. Similarly, another study showed that small-bowel thickening on CT was strongly associated with ICU admission (Wölfel et al., 2020). This relationship did not hold for colon or rectal thickening. These data indicates that small-bowel damage contributes to poor outcomes. As the main organ for nutrient absorption, damage to the small intestine will result in malabsorption and malnutrition, both of which commonly occur in COVID-19 patients (Di Filippo et al., 2021; Lv et al., 2021) and are associated with disease severity (Luo et al., 2020; Zhang P. et al., 2021). A fecal metabolome study showed that feces of COVID-19 patients were enriched with important nutrients that should be metabolized or absorbed, consistent with malabsorption (Lv et al., 2021). A prospective study showed that 29% of COVID-19 patients (31% of hospitalization patients and 21% of patients quarantined at home) had lost >5% of body weight [median weight loss, 6.5 (5.0–9.0) kg or 8.1 (6.1–10.9) %; Di Filippo et al., 2021]. Those patients with weight loss had greater systemic inflammation, impaired renal function and longer disease duration. A large, multicenter study (including 3,229 patients with GI symptoms) showed that 23% of patients had malnutrition, of whom 56.4% were unable to gain weight after 6 months follow-up (Rizvi et al., 2021). Studies also showed that malnutrition was associated with higher incidences of acute respiratory distress syndrome, acute myocardial injury, secondary infection, shock, and 28-day ICU mortality (Luo et al., 2020; Zhang P. et al., 2021). Overall, malabsorption and malnutrition due to damaged small intestine increased disease severity and mortality.

Nutrient absorption in the small intestine is mainly through ATP-dependent active transport. Intestinal infection, hypoxemia, and intestinal ischemia contribute to malabsorption. SARS-CoV-2 adhesion depletes ACE2 levels on intestinal epithelial cells, which alters the expression of the neutral amino acid transporter B0AT1, reducing the intake of tryptophan and the production of nicotinamide (D’Amico et al., 2020). Meanwhile, uncontrolled viral replication consumes large amounts of ATP and nutrients, resulting in decreased nutrients entering the bloodstream. More importantly, anaerobic glycolysis caused by hypoxemia and intestinal ischemia significantly decreases ATP and active transport, leading to malabsorption. Additionally, hypoxemia and intestinal ischemia can also cause anorexia, nausea, vomiting, and enteral nutrition intolerance, reducing food intake. A prospective multicenter study showed that reduced food intake was associated with higher ICU admission and mortality (Caccialanza et al., 2021).

## Intestinal Ischemia and Thrombosis

Intestinal ischemia is a common manifestation in COVID-19 patients. Autopsy results have shown that 31.6% of deceased patients had focal ischemic intestinal changes (Chiu et al., 2020). In a separate imaging study, bowel wall thickening and pneumatosis intestinalis, which indicate intestinal ischemia, were found on 38.1% (16 of 42) of abdominal CT images (Bhayana et al., 2020). Of these, 4 (9.5%) patients with pneumatosis intestinalis developed severe intestinal necrosis and needed resection. In another cohort study, 55.8% (58/104) of ICU patients developed an ileus (Kaafarani et al., 2020). Although mechanical factors cannot be ruled out, insufficient intestinal motility due to intestinal ischemia was more likely to be the cause of ileus in COVID-19 patients. In these patients with ileus, 4 (3.8%) developed severe intestinal ischemia and require emergency surgery. Both studies found microthrombi in these resected intestinal samples, which were the main cause of intestinal ischemia and increased mortality.

Additional intestinal ischemia and necrosis follows the formation of mesenteric thrombosis. However, there is currently relatively little data of mesenteric thrombus in COVID-19. Therefore, we have summarized the characteristics of 40 patients in 39 case reports published on PubMed (Supplementary Table 1). The median age of these patients was 50 (20–82) years, 26 (65%) were male, 38 (95%) developed bowel ischemia or necrosis, 30 (75%) needed bowel resection, 7 (17.5%) required no surgery, at least 3 (7.5%) developed sepsis, and 13 (32.5%) died. Other abdominal thrombotic events (such as celiac aortic thrombosis) leading to mesenteric ischemia can also result in severe intestinal necrosis and require intestinal resection (Zamboni et al., 2021).

**Figure 2:**
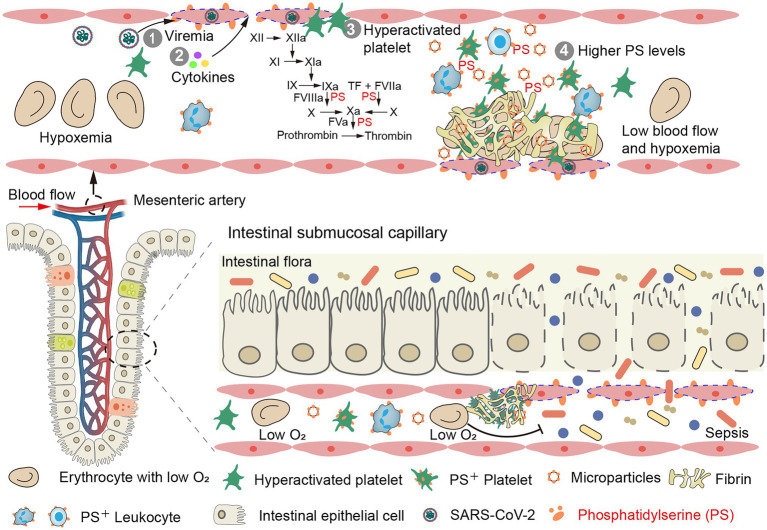
Intestinal thrombosis leads to intestinal mucosal necrosis and dissemination of gut bacteria, endotoxins, and microbial metabolites in blood. **(Top)** Mesenteric vascular endotheliitis (initiated by viremia and accelerated by cytokines), hyperactivated platelets and high levels of phosphatidylserine (PS) promote a high rate of mesenteric thrombus in COVID-19 patients (mesenteric vein is shown in Supplementary Figure 1). **(Bottom)** Intestinal microthrombi and hypoxemia rapidly lead to intestinal mucosal ischemia and necrosis. The damaged gut–blood barrier leads to dissemination of gut bacteria, endotoxins, and microbial metabolites in blood.

Mild intestinal ischemia can lead to reduced diet and malabsorption. Severe intestinal ischemia or necrosis leads to the dissemination of gut bacteria, endotoxins, and microbial metabolites into the blood (Figure 2 bottom), aggravating hyperinflammation and the hypercoagulability state. Such patients need emergency excision of the necrotic bowel, which significantly increases mortality.

## Long-Term Gastrointestinal Sequelae

Long-term GI complications are common in recovering COVID-19 patients. In one systematic review of post-acute COVID-19 manifestations, diarrhea was among the top 10 most common complaints, with a prevalence of 6%. Other long-term GI symptoms include nausea, vomiting, abdominal pain, loss of appetite, and weight loss (Aiyegbusi et al., 2021; Huang et al., 2021). The exact mechanisms of the GI sequelae remain unclear. Recently, persistent endotheliopathy, higher levels of thrombin (Fogarty et al., 2021), and residual SARS-CoV-2 viral antigens in the GI tract (Cheung et al., 2022) were described in convalescent COVID-19 patients. These data suggest that prolonged intestinal infection, persistent endothelial injury (abnormal intestinal–blood barrier), and microthrombi could be causes of the persistent GI symptoms.

## The Mechanisms of Intestinal Thrombosis

### Damaged Endothelial Cells

**Figure 3:**
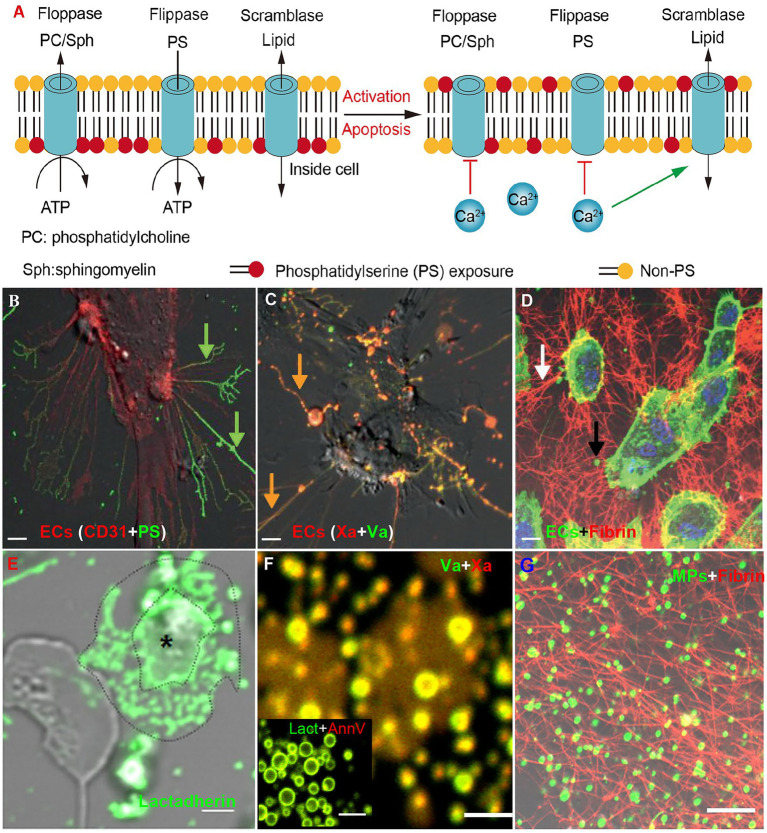
Phosphatidylserine exposure on activated/apoptotic cells and microparticles (MPs) promotes fibrin formation. **(A)** Phosphatidylserine is usually confined to the inner leaflet of the cell membrane. This asymmetry is maintained through ATP-dependent inward transport of PS by flippases and outward transport of non-PS by floppases (left). Upon stimulation, calcium transients will inhibit ATP-dependent transport and stimulate the nonselective lipid transporter scramblase (ATP-independent), resulting in PS exposure (right). **(B–D)** Human umbilical vein ECs were treated with healthy human plasma and TNF-ɑ (our previous study; He et al., 2016). **(B)** ECs retracts the cell margins, extends PS positive filopods and releases endothelial-MPs. **(C)** The PS^+^ filopods and MPs can be co-stained by Xa and Va. **(D)** ECs (green) were incubated with MPs-depleted plasma (MDP) in the presence of calcium for 30 min and stained with Alexa Fluro 647-anti-fibrin for 30 min. Considerable fibrin stands among cultured ECs along with filopodia. **(E)** Confocal images showed PS expression on platelets of patients stained with Alexa 488 lactadherin (our previous study; Ma et al., 2017). MPs from the activated platelet (*) had formed at the margin area located between the distinct outlines. **(F)** MPs from plasma were co-stained by Xa and Va (or lactadherin and annexin V; our previous study; Gao et al., 2015). **(G)** MPs that were incubated with recalcified MDP for 30 min and stained with Alexa Fluro 647-anti-fibrin for 30 min. Converted fibrin networks were detected around MPs. The inset bars represent 5 μm in **(B–D,G)** and 2 μm in **(E,F)**.

Resected bowel samples from COVID-19 patients routinely exhibit thrombi and endotheliitis, indicating the important role of EC injury in mesenteric thrombosis (Bhayana et al., 2020; Chiu et al., 2020; Kaafarani et al., 2020). SARS-CoV-2 infection (Varga et al., 2020) and elevated inflammatory cytokines (He et al., 2016) damage mesenteric vascular ECs. In response, EC cell margins retract, extending phosphatidylserine (PS) positive filopods and releasing endothelial microparticles (MPs; Figure 3B; He et al., 2016). The PS^+^ filopods and MPs can be co-stained by Xa and Va and support fibrin formation (Figures 3B–D). The exposed PS then activates tissue factor on ECs, triggering the extrinsic coagulation pathway (Versteeg et al., 2013). Next, higher levels of FVIII and vWF released from damaged EC contribute to the hypercoagulable state and platelet aggregation, respectively (Goshua et al., 2020). Thrombomodulin is then released from ECs in its soluble form, which has an attenuated capacity to activate Protein C due to a lack of other cofactors on ECs, such as endothelial protein C receptor (Versteeg et al., 2013). Finally, upregulation of endothelial cell adhesion molecules recruits neutrophils and platelets and further contributes to thrombosis (Tong et al., 2020; Li L. et al., 2021).

### Hyperactivated Platelets and Phosphatidylserine Storm

Although COVID-19 patients exhibit mild thrombocytopenia, the remaining platelets are hyperactivated (Manne et al., 2020; Taus et al., 2020; Zaid et al., 2020). Studies have shown that platelets from COVID-19 patients have increased P-selectin and α_IIb_β_3_ expression. P-selectin on activated platelets interacts with integrin α_IIb_/β_3_ on monocytes to form platelet-monocyte complexes, which induce monocyte tissue factor expression (Hottz et al., 2020). The activated platelets can also induce neutrophils to release neutrophil extracellular traps (NETs; Middleton et al., 2020). Furthermore, platelets from COVID-19 patients aggregate and adhere more efficiently to collagen-coated surfaces under flow conditions (Manne et al., 2020; Zaid et al., 2020). Meanwhile, activated platelets release α- and dense-granule contents including FV, FXI, fibrinogen and vWF (Zaid et al., 2020). In addition, activated platelets also produce inflammatory cytokines, fueling cytokine storm (Taus et al., 2020; Zaid et al., 2020). Most importantly, activated platelets expose higher levels of PS and release higher numbers of PS^+^ MPs (Figures 3E–G; Zaid et al., 2020; Althaus et al., 2021).

Phosphatidylserine is the most abundant negatively charged phospholipid in mammalian cells and is usually confined to the inner leaflet of the cell membrane (Versteeg et al., 2013). This asymmetry is maintained through ATP-dependent inward transport of PS by flippases and outward transport of other phospholipids by floppases (Figure 3A left). Upon stimulation, transiently increased calcium inhibits ATP-dependent transport and stimulates the nonselective lipid transporter scramblase (ATP-independent), resulting in PS exposure on the outer membrane (Figure 3A right). During this process, microvesicles derived from the budding of cellular membranes will be released. These MPs are typically <1 μm and express PS (Burnier et al., 2009). The exposure of PS on the surface of cells and MPs provides a catalytic surface for factor Xa and thrombin formation *in vivo* (Versteeg et al., 2013). We have previously demonstrated that PS mediates 90% of Xa and thrombin formation and significantly increases thrombosis *in vivo* (Shi and Gilbert, 2003).

Cytokines and virus infection can activate blood cells and ECs, resulting in higher levels of PS^+^ cells and MPs. As COVID-19 progresses, the developing cytokine storm activates more blood cells, leading to PS storm. Platelets are highly sensitive to circulating cytokines, releasing large amounts of cytokines and PS exposed MPs into the plasma (Taus et al., 2020; Althaus et al., 2021) and thus are a major contributor to PS storm. Previous studies found an unusual elevation of FVa in severe COVID-19 patients (248 IU/dl, higher than any previous disease; Stefely et al., 2020; von Meijenfeldt et al., 2021). The degree of FVa elevation in these patients may be the result of PS storm.

Collectively, SARS-CoV-2 infection is the initiating factor for injury of the intestinal vascular ECs, which is then aggravated by systemic cytokines, leading to endotheliitis. Subsequently, the hyperactivated platelets in circulation rapidly accumulate around the damaged ECs, inducing tissue factor expression, NET release, and activating the intrinsic/extrinsic coagulation pathways. Simultaneously, the high levels of PS expression in circulating cells and MPs further promote thrombin and fibrin formation (Figure 2 top).

## Early Antithrombotic Treatment

Vaccines and antithrombotic therapy are effective measures to reduce intestinal damage and fight against the COVID-19 pandemic (Baden et al., 2021; Chalmers et al., 2021). Vaccines induce adaptive immunity to clear the virus, reducing intestinal infection and intestinal damage. However, the usefulness of vaccines is limited by incomplete vaccine acceptance and viral mutations (Hacisuleyman et al., 2021; Wang et al., 2021). Vaccines are also ineffective for already infected patients. Therefore, more attention should be paid to antithrombotic therapy. Studies had shown that thrombotic events mainly occurred within 7 days of COVID-19 diagnosis (both inpatients and outpatients; Mouhat et al., 2020; Ho et al., 2021). Meanwhile, two large randomized controlled trials (RCTs) from the same platform showed that therapeutic anticoagulation reduced mortality in moderate cases but not in severe ones, suggesting that delayed anticoagulant therapy may lead to treatment failure (REMAP-CAP Investigators et al., 2021a,b). More importantly, a recent study reported three asymptomatic COVID-19 patients who developed abdominal (or intestinal) thrombosis leading to intestinal necrosis (Zamboni et al., 2021). All these data suggest that antithrombotic therapy should be initiated once COVID-19 is diagnosed (excluding patients with contraindications). Early prophylactic antithrombotic therapy can reduce the activation of vascular ECs and blood cells, preventing intestinal thrombosis, ensuring sufficient intestinal perfusion, maintaining the normal gut–blood barrier, avoiding malabsorption, malnutrition, and intestinal flora entering the bloodstream. Further, attenuated injury and decreased microthrombi in convalescent patients may lower the risk of long-term GI sequelae. Meanwhile, unobstructed systemic circulation can also accelerate the removal of SARS-CoV-2, inflammatory cytokines and damaged blood cells by the mononuclear phagocyte system.

### Anticoagulation

Table 1 summarizes the RCTs of anticoagulant therapy in COVID-19 patients. For outpatients, early anticoagulant therapy reduced hospitalization and supplemental oxygen (Gonzalez-Ochoa). While, delayed treatment had no similar effect (ACTIV-4B and Ananworanich). Thus, oral anticoagulant therapy should be initiated in outpatients once COVID-19 is diagnosed. For non-critically ill patients, therapeutic doses of low molecular weight heparin (LMWH) reduced thrombotic events and mortality, and increased organ support-free days (REMAP-CAP, ACTIV-4a, ATTACC; RAPID; HEP-COVID). However, therapeutic doses of rivaroxaban did not improve clinical outcomes and increased bleeding (ACTION). This is potentially because novel oral anticoagulants do not share the anti-inflammatory and antiviral functions of heparin. Intestinal damage might also result in abnormal absorption of oral anticoagulants. Therefore, therapeutic LMWH should be the first choice for non-critically ill patients. For critically ill patients, RCTs showed that moderate and therapeutic doses were not superior to prophylactic ones. Results from several other studies suggest that the overwhelming thrombosis leads to failure of anticoagulant therapy at therapeutic doses (Leentjens et al., 2021; Poor, 2021). Faced with this dilemma, an editorial in *N Engl J Med* argued that profibrinolytic strategies should be considered (Ten Cate, 2021). More studies are needed to explore optimal antithrombotic therapy in critically ill patients.

**Table 1:** Randomized clinical trials of anticoagulant therapy in COVID-19 patients.

	Drugs	Dose/Patients	Interval^§^ (days)	Primary outcomes	Major bleeding^&^
Outpatients					
Connors et al., 2021 (ACTIV-4B)	Apixaban	Control: 164 Prophylactic: 165 Therapeutic: 164	10	AT did not reduce major thromboembolism or death	0 vs. 0 vs. 0
Ananworanich et al., 2021	Rivaroxaban	Control: 222 Prophylactic: 222	<10	AT did not reduce disease progression, but increase asymptomatic participants^*^	0 vs. 0
Gonzalez-Ochoa et al., 2021	Sulodexide	Control: 119 Therapeutic: 124	<3	Fewer patients with AT required hospitalization and supplemental oxygen^*^	0 vs. 1
Non-critically ill patients					
REMAP-CAP Investigators et al., 2021a	LMWH	Prophylactic: 1050 Therapeutic: 1181	<3	Therapeutic AT increased the probability of survival or organ support-free days^*^	0.9% vs. 1.9%
Sholzberg et al., 2021 (RAPID)	LMWH	Prophylactic: 237 Therapeutic: 228	1.5	Mortality (vs. Prophylactic): OR: 0.22 (0.07–0.65)^*^	1.7% vs. 0.9%
Spyropoulos et al., 2021 (HEP-COVID)	Enoxaparin	Prophylactic: 124 Therapeutic: 129	<3	Therapeutic anticoagulation significantly reduced major thromboembolism and death^*^	1.6% vs. 4.7%
Marcos-Jubilar et al., 2022	Bemiparin	Prophylactic: 33 Therapeutic: 32	6 vs. 5^&^	Mortality (vs. Prophylactic): OR: 2.13 (0.18–24.76)	0 vs. 0
Lopes et al., 2021 (ACTION)	Rivaroxaban Enoxaparin^¦^	Prophylactic: 304 Therapeutic: 311	<3	Mortality (vs. Prophylactic): RR: 1.49 (0.90–2.46)	2% vs. 8%^*^
Severe patients					
INSPIRATION Investigators et al., 2021	Enoxaparin	Prophylactic: 276 Intermediate: 286	4	Mortality (vs. Prophylactic): HR: 1.06 (0.83–1.36)	2.5% vs. 1.4%
Perepu et al., 2021	Enoxaparin	Prophylactic: 86 Intermediate: 87	5	Mortality (vs. Prophylactic): OR: 0.66 (0.30–1.45)	2.3% vs. 2.3%
REMAP-CAP Investigators et al., 2021b	LMWH	Prophylactic: 567 Therapeutic: 536	<3	Therapeutic AT did not increase probability of survival or organ support-free days	2.3% vs. 3.8%
Lemos et al., 2020 (HESACOVID)	Enoxaparin	Prophylactic: 10 Therapeutic: 10	<4	Therapeutic AT significantly increased PaO_2_/FiO_2_ ratio	0 vs. 0

* *p* < 0.05.

§ The median time from diagnosis to initiation of study treatment.

& vs. Prophylactic/control.

¦ Clinically stable patients received therapeutic rivaroxaban and clinically unstable ones received therapeutic enoxaparin or unfractionated heparin.

AT, anticoagulation; LMWH, low molecular weight heparin; OR/HR, odds/hazard ratio; and RR, relative risk.

### Inhibition of Platelet Activation

As COVID-19 progresses, cytokine storm activates platelets, which not only participate in primary hemostasis, but also are the major components of PS storm. Autopsy results show a high prevalence of platelet-fibrin-rich microthrombi in lung and extrapulmonary organs, including the gastrointestinal tract (Bradley et al., 2020; Polak et al., 2020). Early inhibition of platelet activation can reduce platelet activity and prevent PS storm, thus decreasing thrombosis and mortality. Several observational studies have shown that aspirin decreases mechanical ventilation, ICU admission, and mortality (Chow et al., 2020; Santoro et al., 2022). The RCTs testing antiplatelet agents were still preliminary. A recent RCT suggested that aspirin was associated with an increase in survival and reduction in thrombotic events (RECOVERY Collaborative Group, 2022). In addition, anti-inflammatory therapy (e.g., dexamethasone, 6 mg once daily; RECOVERY Collaborative Group et al., 2020) inhibits cytokine storm, as well as platelet activation, reducing mortality. Overall, inhibition of platelet activation is also important to reduce mortality through the prevention of thrombosis and organs damage.

## Factors Influencing Antithrombotic Treatment

### Thrombotic Risk Factors or Co-morbidities

Studies have shown that obesity, hyperglycemia and diabetes are associated with increased thrombotic events (including intestinal thrombosis), COVID-19 severity, and mortality (Drucker, 2021; Stefan et al., 2021). Other thrombotic risk factors include previous venous thromboembolism, active cancer, known thrombophilic condition, recent trauma or surgery, age ≥70 years, respiratory/cardiac/renal failure, and inflammatory bowel disease (Susen et al., 2020). These factors or co-morbidities heighten basal inflammatory levels and endothelial damage, leading to premature cytokine and PS storms, ultimately increasing thrombosis and mortality. Thus, more active antithrombotic therapy strategies should be adopted in these patients. For patients with mild COVID-19 with these factors, the French Working Group on Perioperative Hemostasis and the French Study Group on Thrombosis and Hemostasis recommend higher (intermediate) doses of anticoagulant therapy (Susen et al., 2020). For moderately ill patients, therapeutic doses of anticoagulant therapy should be initiated as soon as possible to prevent excessive microthrombus formation. The need for extended thromboprophylaxis in discharged patients remains controversial. However, a recent RCT showed that rivaroxaban (10 mg/day, 35 days) improved clinical outcomes in discharged COVID-19 patients with higher thrombotic risk factors (Ramacciotti et al., 2022), supporting extended thromboprophylaxis in patients with these risk factors or co-morbidities.

### Vaccination

Although more than half the world population has received at least one dose of the vaccines, there are relatively little data of antithrombotic therapy in vaccinated patients. Studies of viral dynamics show that the viral loads of vaccinated patients are as high as that of unvaccinated patients, but drop significantly faster (Brown et al., 2021; Klompas, 2021). Thus, vaccinated patients have shorter hospital stays, and are less likely to progress to critical illness and death (Tenforde et al., 2021; Thompson et al., 2021). Nevertheless, antithrombotic therapy is still beneficial for the vaccinated patients. Firstly, heparin has anti-inflammatory and antiviral functions and can interfere with the binding of SARS-CoV-2 to ACE2 and shorten the duration of virus infection (Kwon et al., 2020; Pereyra et al., 2021). Secondly, antithrombotic therapy protects cells from damage, PS exposure, and microthrombi formation, maintains unobstructed blood circulation, and facilitates virus clearance (by vaccine-induced adaptive immunity). Thirdly, thrombosis remains an important factor in disease progression. Antithrombotic therapy further reduces thrombosis and mortality, especially in vaccinated patients with high risk factors or co-morbidities. Lastly, although vaccines reduce the incidence, a subset of vaccinated patients will still develop long-term sequelae or Long Covid (Ledford, 2021; Antonelli et al., 2022). Persistent viral infection and microthrombi are the primary causes (Ledford, 2021; Xie et al., 2022), and early antithrombotic therapy is still needed to prevent them.

## Conclusion and Future Research

During COVID-19 disease progression, SARS-CoV-2 infiltrates the blood stream from the initial respiratory tract infection, causing viremia, hyperactivated platelets and PS storm. The virus settles into the vascular beds of extrapulmonary organs, ultimately causing infection of intestinal epithelial cell. Damaged ECs, combined with hyperactivated platelets and PS storm, promote intestinal thrombosis, resulting in intestinal ischemia or necrosis. The damaged gut–blood barrier leads to malabsorption, malnutrition and intestinal flora entering the bloodstream, which significantly increase disease severity and mortality. Prolonged intestinal infection, persistent endothelial injury and microthrombi contribute to the long-term GI sequelae after discharge. Early prophylactic antithrombotic therapy can prevent microthrombi, ensuring sufficient intestinal perfusion, maintaining the normal intestinal function, and reducing the risk of long-term GI sequelae. More active antithrombotic therapy should be adopted in patients with other thrombotic risk factors or co-morbidities. Even in vaccinated COVID-19 patients, antithrombotic therapy is also important to decrease (intestinal) thrombosis, mortality and the risk of long-term GI sequelae.

With the Omicron pandemic, patients requiring hospitalization and ICU treatment decline rapidly. However, people are increasingly concerned about Long Covid. In terms of long-term GI sequelae, the detailed mechanisms of prolonged intestinal infection and persistent microthrombi remain unclear. And whether anticoagulant therapy can decrease GI symptoms in patients with long-term GI sequelae deserves further study. Finally, the impact of vaccines on long-term GI sequelae remains unclear in previously infected and breakthrough infected patients.

## Author Contributions

XW, TJ, and JS designed the research. XW and JS analyzed the data and wrote the primary manuscript. HJ, CW, YW, NZ, and TJ contributed to literature retrieval, data acquisition, and analysis of data. VN analyzed the data and revised the primary manuscript. JS and VN discussed and confirmed the final manuscript. All authors contributed to the article and approved the submitted version.

## Conflict of Interest

The authors declare that the research was conducted in the absence of any commercial or financial relationships that could be construed as a potential conflict of interest.

The reviewer (UDA) declared a shared affiliation with the authors (VN and JS) to the handling editor at the time of review.

## Publisher’s Note

All claims expressed in this article are solely those of the authors and do not necessarily represent those of their affiliated organizations, or those of the publisher, the editors and the reviewers. Any product that may be evaluated in this article, or claim that may be made by its manufacturer, is not guaranteed or endorsed by the publisher.

## Supplementary Material

The Supplementary Material for this article can be found online at: https://www.frontiersin.org/articles/10.3389/fmicb. 2022.860931/full#supplementary-material

## Abbreviations

COVID-19: Coronavirus disease 2019
SARS-CoV-2: Severe acute respiratory syndrome
ACE2: Angiotensin-converting enzyme 2
GI: Gastrointestinal
ECs: Endothelial cells
vWF: von Willebrand factor
PS: Phosphatidylserine
MPs: Microparticles
TF: Tissue factor
NETs: Neutrophil extracellular traps
RCT: Randomized controlled trial
LMWH: Low molecular weight heparin
RT-PCR: Reverse transcription-polymerase chain reaction
