# Supplementary material for: Intestinal Damage in COVID-19: SARS-CoV-2 Infection and Intestinal Thrombosis
Source: Front Microbiol. 2022 Mar 22;13:860931. doi: 10.3389/fmicb.2022.860931 (PMC8981312; doi:10.3389/fmicb.2022.860931)
Supplement: Supplementary file 1 [file Data_Sheet_1.PDF]

## Supplementary Material

### Methods

*COVID-19 patients with mesenteric thrombosis*

We searched PubMed for the data of COVID-19 patients with mesenteric thrombosis using the search terms "COVID-19", "SARS-CoV-2", "mesenteric", "thrombosis", "mesenteric ischemia", "bowel ischemia" and "intestinal ischemia". All case reports that included COVID-19 associated mesenteric thrombosis (last updated on November 4, 2021) were reviewed. Cases without mesenteric thrombus, letters, comments, and systematic reviews were excluded. Cohort studies of thrombotic complications in COVID-19 were also excluded due to a lack of detailed data of patients with mesenteric thrombosis.

*Randomized clinical trials of anticoagulant therapy in COVID-19 patients.*

Using search terms "COVID-19", "SARS-CoV-2", "anticoagulation", "heparin", "enoxaparin", "direct oral anticoagulant", "rivaroxaban" and "Sulodexide", we searched PubMed for randomized clinical trials of anticoagulant therapy in COVID-19 patients.

### Supplementary Figure

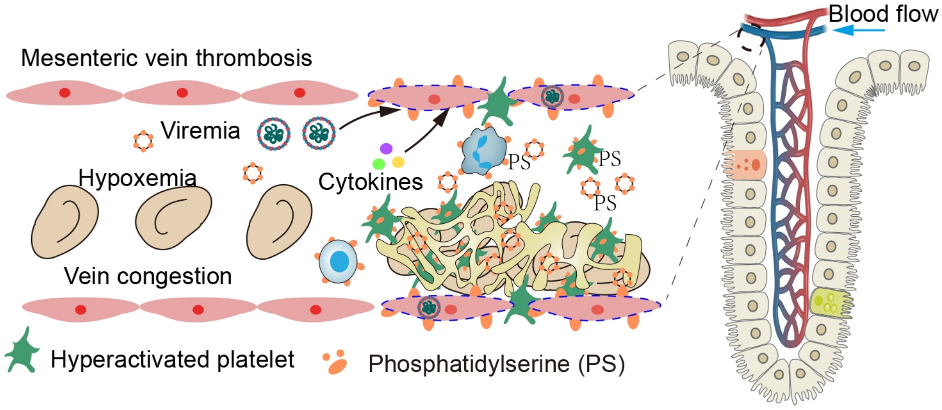


**Supplementary Figure 1 Mesenteric vein thrombosis after COVID-19 pneumonia.** Mesenteric vascular endotheliitis (initiated by viremia and accelerated by cytokines), hyperactivated platelets and high levels of phosphatidylserine promote a high rate of mesenteric thrombosis in COVID-19 patients.

**Supplemental Table 1** Characteristics of COVID-19 patients with mesenteric thrombosis.

| | Age | Gender | Thrombosis | Intestinal tract | Surgery | Death |
|---|---|---|---|---|---|---|
| Mitchell JM et al | 69 | M | SMA | Mucosal necrosis | Resection | |
| Fan BE et al | 30 | M | MV | Intestine obstruction | Resection | |
| Pang JHQ et al | 30 | M | SMV | Jejunum narrow | Resection | |
| Vulliamy P et al | 60 | M | DA/SMA | Bowel necrosis | Resection | |
| Rodriguez et al | 45 | M | SMA | Bowel necrosis | Resection/ | |
| Rodriguez et al | 42 | F | MV | Bowel perforation | Laparotomy [§] | √[*] |
| Cheung S et al | 55 | M | SMA | Bowel necrosis | Resection | |
| de Barry O et al | 79 | F | PV/SMV/SMA | Bowel necrosis | Resection | √ |
| Gartland RM et al | 47 | M | -- | Bowel perforation | Laparotomy [§] | √ |
| English W et al | 40 | M | -- | Bowel necrosis | Resection | |
| Farina D et al | 70 | M | SMA | Bowel ischemia | No | √ |
| Chiu CY et al | 49 | F | MA | Jejunum necrosis | Resection (59 cm) | |
| Carmo FA et al | 33 | M | IMV | Back pain | No (Heparin) | |
| Karna ST et al | 51 | F | SMA | Bowel perforation | Resection | √ |
| Azouz E et al | 56 | -- | SMA | Bowel necrosis | Resection (2 m) | |
| A Beccara L et al | 52 | M | SMA | Bowel ischemia | Resection | |
| Norsa L et al | 62 | M | IV/SMV | Bowel ischemia | Resection | √[*] |
| Lari E et al | 38 | M | -- | Bowel ischemia | Resection | |
| Ignat M et al | 28 | F | SMV | Jejunal ischemia | Resection (80 cm) | |
| Stahl K et al | 43 | M | MV | Bowel necrosis | Hemicolectomy | |
| Besutti G et al | 72 | M | DA/SMA | Bowel ischemia | Resection | |
| Goodfellow et al | 36 | F | SMV | Bowel ischemia | No (Heparin) | |
| Sehhat S et al | 77 | M | MV | Bowel necrosis | Resection | √ |
| Ferraris LE et al | 65 | F | SMA | Bowel ischemia | Resection | √ |
| Singh B et al | 82 | F | MA | Gangrenous colon | Ileostomy | |
| Alemán W et al | 44 | M | MV | Bowel ischemia | No (Heparin) | |
| Osilli D et al | 75 | M | SMA | Gangrenous Ileum | Resection (30 cm) | - |
| Kiwango F et al | 60 | F | MV | Bowel necrosis | No | √ |
| Al Mahruqi G et al | 51 | M | SMA | Gangrenous jejunum | Resection | |
| Mir MZ et al | 59 | F | MV | Bowel necrosis | No | √ |
| Hanif M et al | 20 | F | SMA | Bowel necrosis | Resection | |
| Bannazadeh et al | 45 | M | SMA | Ileum necrosis | Resection/Heparin | |
| Amaravathi et al | 45 | M | SMA/SMV | Bowel necrosis | Resection (103 cm) | - |
| Ucpinar BA et al | 82 | F | SMA | -- | -- | √ |
| Thuluva SK et al | 29 | M | SMV | Bowel ischemia | No (Heparin) | |
| Balani P et al | 37 | M | SMA | Bowel ischemia | No (Thrombolysis) | |
| Calcagno E et al | 36 | M | PV/SMV | Ileum necrosis | Resection | |
| Ebrahiminik et al | 35 | M | PV/SMV | Bowel ischemia | No (Thrombolysis) | |
| Jain M et al | 57 | F | MV | Bowel necrosis | Resection | √ |
| Zamboni P et al | 64 | F | IMV | Bowel ischemia | Sigmoidectomy | √[*] |

[*] patients developed septic shock; [§] bowel resection was not performed due to critically ill status; M, male; F, female; SMA/MA, (superior) mesenteric artery; SMV/IMV/MV, (superior)/(inferior) mesenteric vein; PV, portal vein; DA, descending aorta; IV, Inferior vena

## Supplemental references


A Beccara, L., Pacioni, C., Ponton, S., Francavilla, S., Cuzzoli, A. (2020). Arterial mesenteric thrombosis as a complication of SARS-CoV-2 infection. *Eur. J. Case Rep. Intern. Med.* 7, 001690. doi: 10.12890/2020_001690

Al Mahruqi, G., Stephen, E., Abdelhedy, I., Al Wahaibi, K. (2021). Our early experience with mesenteric ischemia in COVID-19 positive patients. *Ann. Vasc. Surg.* 73, 129-132. doi: 10.1016/j.avsg.2021.01.064

Alemán, W., Cevallos, L. C. (2021). Subacute mesenteric venous thrombosis secondary to COVID-19: A late thrombotic complication in a nonsevere patient. *Radiol. Case Rep.*16, 899-902. doi: 10.1016/j.radcr.2021.01.039

Amaravathi, U., Balamurugan, N., Muthu Pillai, V., Ayyan, S. M. (2021). Superior mesenteric arterial and venous thrombosis in COVID-19. *J. Emerg. Med.* 60, e103-e107. doi: 10.1016/j.jemermed.2020.12.016

Azouz, E., Yang, S., Monnier-Cholley, L., Arrivé, L. (2020). Systemic arterial thrombosis and acute mesenteric ischemia in a patient with COVID-19. *Intensive Care Med.* 46, 1464-1465. doi: 10.1007/s00134-020-06079-2

Balani, P., Bhuiyan, A. S., Dalal, V. N., Maheshwari, G. S. (2021). Early detection and successful management of acute mesenteric ischaemia in symptomatic COVID-19 patient. *Indian J. Surg.* doi: 10.1007/s12262-021-02839-6.

Bannazadeh, M., Tassiopoulos, A., Koullias, G. (2021). Acute superior mesenteric artery thrombosis seven days after discharge for novel coronavirus pneumonia (NCP). *J. Vasc. Surg. Cases Innov. Tech.* 7, 586-588. doi: 10.1016/j.jvscit.2020.12.002

Besutti, G., Bonacini, R., Iotti, V., Marini, G., Riva, N., Dolci, G., et al. (2020). Abdominal visceral infarction in 3 patients with COVID-19. *Emerg. Infect. Dis.* 26, 1926-1928. doi: 10.3201/eid2608.201161

Calcagno, E., Sogunro, O., Nepal, P., Assaker, R., Sapire, J. (2021). COVID-19 induced mesenteric venous infarction. *Radiol. Case Rep.* 16, 1999-2002. doi: 10.1016/j.radcr.2021.04.083

Carmo Filho, A., Cunha, B. D. S. (2020). Inferior mesenteric vein thrombosis and COVID-19. *Rev. Soc. Bars. Med. Trop.* 53, e20200412. doi: 10.1590/0037-8682-0412-2020

Cheung, S., Quiwa, J. C., Pillai, A., Onwu, C., Tharayil, Z. J., Gupta, R. (2020). Superior mesenteric artery thrombosis and acute intestinal ischemia as a consequence of COVID-19 infection. *Am. J. Case Rep.* 21, e925753. doi: 10.12659/AJCR.925753

Chiu, C. Y., Sarwal, A., Mon, A. M., Tan, Y. E., Shah, V. (2021). Gastrointestinal: COVID-19 related ischemic bowel disease. *J. Gastroenterol. Hepatol.* 36, 850. doi: 10.1111/jgh.15254

de Barry, O., Mekki, A., Diffre, C., Seror, M., El Hajjam, M., Carlier, R. Y. (2020). Arterial and venous abdominal thrombosis in a 79-year-old woman with COVID-19 pneumonia. *Radio. Case Rep.* 15, 1054-1057. doi: 10.1016/j.radcr.2020.04.055

Ebrahiminik, H., Nikpour, S., Yazdi, H. R., Mohammadi, A., Mirza-Aghazadeh-Attari, M. (2021). Successful vascular interventional management of superior mesenteric vein thrombosis in a patient with COVID-19: A case report and review of literature. *Radiol. Case Rep.* 16, 1539-1542. doi: 10.1016/j.radcr.2021.03.038

English, W., Banerjee, S. (2020). Coagulopathy and mesenteric ischaemia in severe SARS-CoV-2 infection. *ANZ J. Surg.* 90, 1826. doi: 10.1111/ans.16151

Fan, B. E., Chang, C. C. R., Teo, C. H. Y., Yap, E. S. (2020). COVID-19 Coagulopathy with superior mesenteric vein thrombosis complicated by an ischaemic bowel. *Hamostaseologie* 40, 592-593. doi: 10.1055/a-1232-7446

Farina, D., Rondi, P., Botturi, E., Borghesi, A., Guelfi, D., Ravanelli, M. (2020;). Gastrointestinal: Bowel ischemia in a suspected coronavirus disease (COVID-19) patient. *J. Gastroenterol. Hepatol.* 36, 41. doi: 10.1111/jgh.15094

Ferraris, L. E., Sala, G., Casalino, S., Losurdo, L., De Filippis, V. (2020). Mesenteric artery thrombosis, microvascular intestinal endothelitiis, and Guillain-Barrè Syndrome in the same SARS-CoV-2 patient. *Cureus.* 12, e11326. doi: 10.7759/cureus.11326

Gartland, R. M., Velmahos, G. C. (2020). Bowel Necrosis in the Setting of COVID-19. *J. Gastrointest. Surg.* 24, 2888-2889. doi: 10.1007/s11605-020-04632-4

Goodfellow, M., Courtney, M., Upadhyay, Y., Marsh, R., Mahawar, K. (2021). Mesenteric venous thrombosis due to coronavirus in a post Roux-en-Y gastric bypass patient: a case report. *Obes. Surg.* 8, 1-3. doi: 10.1007/s11695-020-05214-8

Hanif, M., Ahmad, Z., Khan, A. W., Naz, S., Sundas, F. (2021). COVID-19-induced mesenteric thrombosis. *Cureus.* 13, e12953. doi: 10.1016/j.radcr.2021.04.083

Ignat, M., Philouze, G., Aussenac-Belle, L., Faucher, V., Collange, O., Mutter, D., Pessaux, P. (2020). Small bowel ischemia and SARS-CoV-2 infection: an underdiagnosed distinct clinical entity. *Surgery* 168, 14-16. doi: 10.1016/j.surg.2020.04.035

Jain, M., Tyagi, R., Tyagi, R., Jain, G. (2021). Post-COVID-19 gastrointestinal invasive mucormycosis. *Indian J. Surg.* doi: 10.1007/s12262-021-03007-6.

Karna, S. T., Panda, R., Maurya, A. P., Kumari, S. (2020). Superior mesenteric artery thrombosis in COVID-19 pneumonia: an underestimated diagnosis-first case report in Asia. *Indian J. Surg.* doi: 10.1007/s12262-020-02638-5.

Kiwango, F., Mremi, A., Masenga, A., Akrabi, H. (2021). Intestinal ischemia in a COVID-19 patient: case report from Northern Tanzania. *J. Surg. Case Rep.* 1, rjaa537. doi: 10.1093/jscr/rjaa537

Lari, E., Lari, A., AlQinai, S., Abdulrasoul, M., AlSafran, S., Ameer, A., Al-Sabah, S. (2020). Severe ischemic complications in Covid-19-A case series. *Int. J. Surg. Case Rep.* 75, 131-135. doi: 10.1016/j.ijscr.2020.09.009

Mir, M. Z., Mashhadi, A., Jahantigh, M., Seyedi, S. J. (2020). Bowel necrosis associated with COVID-19 pneumonia: A report of two cases. *Radiol. Case Rep.* 16, 819-23. doi: 10.1016/j.radcr.2021.01.043

Mitchell, J. M., Rakheja, D., Gopal, P. (2021). SARS-CoV-2-related hypercoagulable state leading to ischemic enteritis secondary to superior mesenteric artery thrombosis. *Clin. Gastroenterol. Hepatol.* 19, e111. doi: 10.1016/j.cgh.2020.06.024

Norsa, L., Valle, C., Morotti, D., Bonaffini, P. A., Indriolo, A., Sonzogni, A. (2020). Intestinal ischemia in the COVID-19 era. *Dig. Liver Dis.* 52, 1090-1091. doi: 10.1016/j.dld.2020.05.030

Osilli, D., Pavlovica, J., Mane, R., Ibrahim, M., Bouhelal, A., Jacob, S. (2020). Case reports: mild COVID-19 infection and acute arterial thrombosis. *J. Surg. Case Rep.* 9, rjaa343. doi: 10.1093/jscr/rjaa343

Pang, J. H. Q., Tang, J. H., Eugene-Fan, B., Lee, C. L., Low, J. K. (2020). A peculiar case of small bowel stricture in a coronavirus disease 2019 patient with congenital adhesion band and superior mesenteric vein thrombosis. *Ann. Vasc. Surg.* 70, 286-289. doi: 10.1016/j.avsg.2020.08.084

Rodriguez-Nakamura, R. M., Gonzalez-Calatayud, M., Martinez, Martinez, A. R. (2020). Acute mesenteric thrombosis in two patients with COVID-19. Two cases report and literature review. *Int. J. Surg. Case Rep.* 76, 409-414. doi: 10.1016/j.ijscr.2020.10.040

Sehhat, S., Talebzadeh, H., Hakamifard, A., Melali, H., Shabib, S., Rahmati, A., et al. (2020). Acute Mesenteric Ischemia in a Patient with COVID-19: A Case Report. *Arch. Iran. Med.* 23, 639-643. doi: 10.34172/aim.2020.77

Singh, B., Mechineni, A., Kaur, P., Ajdir, N., Maroules, M., Shamoon, F., Bikkina, M. (2020). Acute Intestinal Ischemia in a Patient with COVID-19 Infection. *Korean J. Gastroenterol.* 76, 164-166. doi: 10.4166/kjg.2020.76.3.164

Stahl, K., Bräsen, J. H., Hoeper, M. M., David, S. (2020). Direct evidence of SARS-CoV-2 in gut endothelium. *Intensive Care Med.* 46, 2081-2082. doi: 10.1007/s00134-020-06237-6

Thuluva, S. K., Zhu, H., Tan, M. M. L., Gupta, S., Yeong, K. Y., Cheong Wah, S. T, et al. (2020). A 29-Year-Old Male Construction Worker from India Who Presented with Left- Sided Abdominal Pain Due to Isolated Superior Mesenteric Vein Thrombosis Associated with SARS-CoV-2 Infection. *Am. J. Case Rep.* 21, e926785. doi: 10.12659/AJCR.926785

Ucpinar, B. A., Sahin, C. (2020). Superior mesenteric artery thrombosis in a patient with COVID-19: A unique presentation. *J. Coll. Physicians Surg. Pak.* 30, 112-114. doi: 10.29271/jcpsp.2020.supp2.112

Vulliamy, P., Jacob, S., Davenport, R. A. (2020). Acute aorto-iliac and mesenteric arterial thromboses as presenting features of COVID-19. *Br. J. Haematol.* 189, 1053-1054. doi: 10.1111/bjh.16760

Zamboni, P., Bortolotti, D., Occhionorelli, S., Traina, L., Neri, L. M., Rizzo, R., et al. (2021). Bowel ischemia as onset of COVID-19 in otherwise asymptomatic patients with persistently negative swab. *J. Intern. Med.* doi: 10.1111/joim.13385.
